# Testing Surgical Face Masks in an Emergency Context: The Experience of Italian Laboratories during the COVID-19 Pandemic Crisis

**DOI:** 10.3390/ijerph18041462

**Published:** 2021-02-04

**Authors:** Francesco Tessarolo, Giandomenico Nollo, Devid Maniglio, Marta Rigoni, Luca Benedetti, Fabrizia Helfer, Ivan Corradi, Luigi Rovati, Alberto Ferrari, Mattia Piccini, Luca Accorsi, Elena Veronesi, Aurora Cuoghi, Salvo Baglio, Nunzio Tuccitto, Stefania Stefani, Stefano Stracquadanio, Filippo Caraci, Antonio Terrasi, Alessia Tricomi, Mario Musumeci, Andrea Miraglia, Giacomo Cuttone, Sofia Cosentino, Carlo Muscas, Luca Agostino Vitali, Dezemona Petrelli, Leopoldo Angrisani, Roberta Colicchio, Andrea D’Anna, Ivo Iavicoli, Gianluigi De Falco, Francesco Di Natale, Ernesto Di Maio, Paola Salvatore, Fabiana Quaglia, Marina Mingoia, Paolo Castellini, Paolo Chiariotti, Serena Simoni, Luigi Montalto, Alessia Baleani, Nicola Paone

**Affiliations:** 1 LASS-TN-Covid-19 Laboratorio Associato per la Verifica di Dispositivi di Protezione, Dipartimento di Ingegneria Industriale, Università di Trento and Laboratorio di Sanità Pubblica, Azienda Provinciale per i Servizi Sanitari di Trento, 38123 Trento, Italy; giandomenico.nollo@unitn.it (G.N.); devid.maniglio@unitn.it (D.M.); marta.rigoni@unitn.it (M.R.); luca.benedetti@unitn.it (L.B.); fabrizia.helfer@apss.tn.it (F.H.); ivan.corradi@apss.tn.it (I.C.); 2 Dipartimento di Ingegneria Enzo Ferrari, Università di Modena e Reggio Emilia, Modena—Italy and Science & Technology Park for Medicine, TPM, 41037 Modena, Italy; luigi.rovati@unimore.it (L.R.); alberto.ferrari@tpm.bio (A.F.); mattia.piccini@unimore.it (M.P.); luca.accorsi@tpm.bio (L.A.); elena.veronesi@tpm.bio (E.V.); aurora.cuoghi@tpm.bio (A.C.); 3 AntiCovidLab, Torre Biologica, Università degli Studi di Catania, 95123 Catania, Italy; salvatore.baglio@unict.it (S.B.); nunzio.tuccitto@unict.it (N.T.); stefania.stefani@unict.it (S.S.); s.stracquadanio@unict.it (S.S.); fcaraci@unict.it (F.C.); antonio.terrasi@ct.infn.it (A.T.); alessia.tricomi@ct.infn.it (A.T.); musumeci@lns.infn.it (M.M.); miraglia@lns.infn.it (A.M.); cuttone@lns.infn.it (G.C.); 4 INFN-Laboratori Nazionali del Sud-Italia-Sicilia-Catania, 95123 Catania, Italy; 5 Dipartimento di Scienze Mediche e Sanità Pubblica, Università degli Studi di Cagliari, 09124 Cagliari, Italy; scosenti@unica.it (S.C.); carlo.muscas@unica.it (C.M.); 6 UNICAM- U-TYM Lab Microbiologia—ex Dip. Biologia, Università di Camerino, 62032 Camerino, Italy; luca.vitali@unicam.it (L.A.V.); dezemona.petrelli@unicam.it (D.P.); 7 Presidio Tecnico/Scientifico di Ateneo per l’Emergenza COVID-19, Centro Servizi Metrologici e Tecnologici Avanzati, Università di Napoli Federico II, 80124 Naples, Italy; angrisan@unina.it (L.A.); roberta.colicchio@unina.it (R.C.); anddanna@unina.it (A.D.); ivo.iavicoli@unina.it (I.I.); gianluigi.defalco@unina.it (G.D.F.); francesco.dinatale@unina.it (F.D.N.); ernesto.dimaio@unina.it (E.D.M.); paola.salvatore@unina.it (P.S.); fabiana.quaglia@unina.it (F.Q.); 8 LABC19 Centro di Ricerca e Servizio per l’Emergenza COVID-19, Università Politecnica delle Marche, 60121 Ancona, Italy; m.mingoia@staff.univpm.it (M.M.); p.castellini@staff.univpm.it (P.C.); p.chiariotti@staff.univpm.it (P.C.); s.simoni@pm.univpm.it (S.S.); l.montalto@pm.univpm.it (L.M.); a.baleani@pm.univpm.it (A.B.); n.paone@staff.univpm.it (N.P.)

**Keywords:** masks, Covid-19, Sars-2, standard testing, pandemic, bacterial filtration efficiency, differential pressure, third mission

## Abstract

The first wave of the COVID-19 pandemic brought about a broader use of masks by both professionals and the general population. This resulted in a severe worldwide shortage of devices and the need to increase import and activate production of safe and effective surgical masks at the national level. In order to support the demand for testing surgical masks in the Italian context, Universities provided their contribution by setting up laboratories for testing mask performance before releasing products into the national market. This paper reports the effort of seven Italian university laboratories who set up facilities for testing face masks during the emergency period of the COVID-19 pandemic. Measurement set-ups were built, adapting the methods specified in the EN 14683:2019+AC. Data on differential pressure (DP) and bacterial filtration efficiency (BFE) of 120 masks, including different materials and designs, were collected over three months. More than 60% of the masks satisfied requirements for DP and BFE set by the standard. Masks made of nonwoven polypropylene with at least three layers (spunbonded–meltblown–spunbonded) showed the best results, ensuring both good breathability and high filtration efficiency. The majority of the masks created with alternative materials and designs did not comply with both standard requirements, resulting in suitability only as community masks. The effective partnering between universities and industries to meet a public need in an emergency context represented a fruitful example of the so-called university “third-mission”.

## 1. Introduction

The World Health Organization (WHO), on the basis of the vast literature spanning throughout the last century, recognizes that “*The use of masks is part of a comprehensive package of the prevention and control measures that can limit the spread of certain respiratory viral diseases, including COVID-19. Masks can be used either for protection of healthy persons (worn to protect oneself when in contact with an infected individual) or for source control (worn by an infected individual to prevent onward transmission)*” [1].

Masks are specifically designed to cover the nose and mouth, acting as a barrier to limit the spread and infection by microorganisms by aerosols and droplets, thus they are well suited to fight against diseases that spread through respiration. They can be divided into respirators, designed to protect the wearer by filtering droplets and particles down to the tenths of micrometers scale, and surgical masks, designed to prevent the wearer from spreading large droplets (typically > 3 µm). Existing standards provide requirements on minimum performance and consider the wearer to be a worker. No specific standards existed for the use by people not involved in hazardous operations or not working in the healthcare sector. Only in June 2020 was a CEN workshop agreement drafted and approved on “Community face coverings—Guide to minimum requirements, methods of testing and use” [2].

In Western countries, the use of masks, until 2019, was traditionally limited to personnel operating in hospitals and in healthcare settings. On the other hand, in the Eastern countries, the use of masks by the general population has constantly increased in recent decades, due to a growing awareness about personal protection from both pollutants and infectious agents. There, it was more and more frequent to see people wearing masks in crowded areas, such as airports and subways. Evidence on the efficacy of wearing masks by the general population was recently recalled in the ECDC document [3], which states that “*face masks have been used extensively in the public in Asian countries and have been linked to a slightly lower risk of SARS among persons without known contact with SARS patients during the 2003 SARS epidemic*”.

The COVID-19 pandemic brought about a renewed interest in masks and a broader use of them, especially surgical masks, that were introduced as a means to limit the pandemic spread within workers and the population in general. In clinical settings, the use of surgical masks is mainly intended to protect the patient from infectious agents and in specific circumstances, to protect the wearer from splashes of potentially contaminated liquids. In the community, medical face masks are intended to reduce the risk of infections spreading from/to each other and improve individual protection.

### 1.1. The Covid-19 Outbreak in Italy and the Emergency Context

After being initially confined to China, the first two months of 2020 saw COVID-19 being spread to Europe and WHO declared the COVID-19 outbreak as a pandemic in early March 2020.

Italy was the first European country to be severely affected by the disease. The calendar of events evolved rapidly. After the first reported cases on 30 January 2020, involving tourists from China, Italy was the first European country to cancel all direct flights to and from China. Once the first internal outbreak was defined, the quarantine of 11 municipalities in northern Italy (all located in Lombardia and Veneto regions) was imposed by the government. On 23 February 2020, the Council of Ministers issued the decree-law no. 6, which sanctioned the lockdown of the quarantined municipalities. In the following days, a series of implementing Decrees of the President of the Council of Ministers (DPCMs) were issued, in which the restriction measures became increasingly strict [4] and ended up with the lockdown of the whole national territory on 9 March 2020 (so-called “Phase 1”) [5].

With the DPCM of 17 May 2020, “Phase 2” was initiated, lasting from 18 May to 14 June 2020 [5]. During this period, most of activities were permitted and some restrictions, such as social isolation and regional confinement, were removed. After 3 June 2020, citizens were allowed to move again throughout the country (“Phase 3”). Since decree 23 February 2020, the use of masks was enforced in all public access indoor environments.

### 1.2. The Market Dynamics of Surgical Face Masks and the Request to Set Up Testing Laboratories at Italian Universities

An immediate and direct consequence of the pandemic was the sudden increase in the demand for personal protective equipment (PPE). This quickly led to a worldwide shortage of PPE, especially face masks. The increase in demand for PPE was sustained by panic reaction in the population, in a context of misinformation as well as stockpiling. This situation was clearly described in [3] and in Italy led to a severe shortage of surgical masks for healthcare workers. On 6 April 2020, WHO issued the recommendation document about a “*Rational use of individual protection for coronavirus disease (COVID-19) and considerations for severe deficiencies*”, addressing the problem of PPE shortage and proposing temporary emergency measures including prolonged use of respirators or the use of alternative mask models, including surgical masks when respirators were not available. The document also assessed the disruption of the global supply chain and drafted considerations for decision making processes during major shortages of PPE [6,7].

In this context, while the need for face masks and the requests by healthcare professionals were increasing, the population’s demand was chaotic, due to a lack of specific knowledge, competence and regulations. The generic term “*mask*” was among the most widely used on social media and health communication channels [8,9]. The term “*community mask*” became familiar to define nonmedical masks [3], including various forms of self-made or commercial masks made of cloth or other fabrics and materials—for example, paper. Community masks were not standardized and were not designed for use by healthcare professionals.

Among the measures undertaken to counteract the emergency, the Italian Government issued specific decrees regarding PPE [4,5]. Two DPCMs opened up the possibility to use, import or produce and distribute PPE without the CE mark (conformity with health, safety, and environmental protection standards for products sold within the European Economic Area) in Italy. However, producers or importers were asked to declare the conformity of the devices to existing safety and efficacy standards.

The Istituto Superiore di Sanità (ISS), in the role of technical-scientific body of the National Health Service in Italy, was in charge of checking the documentation and test reports presented by producers or importers of surgical masks, according to the procedures described in [10]. The approval process required surgical masks to satisfy the minimum requirements for safety and efficacy specified in the EN 14683:2019+AC [11] and the biocompatibility requirements specified in the ISO 10993-1:2010 [12]. For community masks, no specific requirement applied at that time.

In the first week of March 2020, in order to support the rapidly emerging demand for testing essential requirements of surgical masks, the Ministry of University invited the Italian universities to set up laboratories able to perform mask testing.

This paper describes how seven Italian universities responded to this request and brought into operation, in a short timeframe, new laboratory facilities and procedures to meet producers’ testing requests, measurement specifications and reporting activities. A large amount of data about the two main performance indicators, Differential Pressure (DP) and Bacterial Filtration Efficiency (BFE), were included in this paper in order to present the characteristics of the various prototypes and products tested in relation to standard requirements. The strategies adopted and limitations encountered in carrying out the testing activities in the emergency context are also discussed.

## 2. Materials and Methods

### 2.1. The Standard EN 14683:2019+AC

The European Standard EN 14683:2019+AC “*Medical face masks—Requirements and test methods*” [11] specifies performance requirements and test methods for medical face masks. The standard indicates the minimum values for BFE, the maximum values for DP (related to breathability), the maximum bioburden (indicating the microbial cleanliness) and, for specific applications, splash resistance requirements (in case the user is exposed to the risk of body fluid spillage). In this paper, only the results for DP and BFE were included.

The same standard identifies three different types of surgical masks (Type I, Type II and Type IIR) according to the DP and BFE thresholds indicated in Table 1.

Annex B of the standard [11] describes the test method for BFE. Briefly, an aerosol stream containing a known charge of *Staphylococcus aureus* ATCC 6538 is forced to pass through the mask material and a multiple stage cascade impactor [13]. The most relevant specifications for the test rig are the following:

- the aerosol should be mixed with air in a cylinder with an 80 mm diameter and 600 mm length;
- the air flow in the system should be generated and kept constant by a vacuum pump at a rate of 28.3 L/min;
- the aerosol should be obtained from a bacterial suspension grown in tryptic soy broth and then diluted in peptone water up to a concentration of 5 × 10^5^ CFU/mL (CFU stands for Colony Forming Units);
- the Mean Particle Size (MPS) of the aerosol should be in the range from 2.7 µm to 3.3 µm;
- the average number of CFUs in the two positive controls (tests without mask) should be between 1700 and 3000.

A six-stage Andersen impactor [14] should be used. Each of the six stages consists of 400 orifices and a Petri dish, containing an agar culture medium, used as impaction plates. Depending on orifices’ diameters, droplets of a given class-size impact on the Petri dish and trigger the formation of a colony of bacteria. After the sampling period is completed, the plates should be incubated, and colonies enumerated using the “positive hole” correction for stages 3–6, as described by Andersen [14]. The number of CFUs for each plate provides the size distribution of the aerosol droplets. The Mean Particle Size (MPS) of the generated aerosol should be measured for each testing session according to the specifications of the impactor [11] and the following formula:

(1) $$MPS=\frac{\sum_{i=1}^{6}\left(P_{i}\times C_{i}\right)}{\sum_{i=1}^{6}C_{i}}$$

where *P_i_* is the particle diameter having a 50% probability of being captured by the *i*-th stage of the impactor, and *C_i_* is the number of CFUs grown at the *i*-th stage when no mask is present in the system.

The bacterial filtration efficiency, expressed as a percentage, is given by the number of CFUs that pass through the material (*CFU_test_*) of the face mask with respect to the number of CFUs of the incoming aerosol (*CFU_control_*), according to the following formula [11]:

(2) $$BFE\;\left(\%\right)=\frac{CFU_{control}-CFU_{test}}{CFU_{control}}\times 100$$

In order to evaluate the number of CFUs of the incoming aerosol, a positive test without mask is required. The method also requires the execution of a negative test performed without the generation of the bacterial aerosol, which should show no CFU growth. BFE should be measured on at least 5 samples and the mean value should be compared to the requirements.

The standard does not explicitly require an estimate of the uncertainty of the measurement; however, it suggests calibrating the flowmeter. It is worth noting that, to discriminate between BFE 95 or 98%, the uncertainty of the BFE measurement should be significantly lower than 1%, according to the generally recognized criterion that in conformance assessments the ratio of tolerance to measurement uncertainty should be in the range from 4 to 10 [15].

Annex C of the standard [11] provides the test method specifications for the measurement of the DP. It defines specifications to make a testing system able to force a constant air flow (8 L/min) through a circular section of the mask having a (25 ± 1) mm diameter. The DP between the two surfaces of the mask sample was measured with a differential manometer. The DP has to be measured on at least 5 masks and for each mask 5 different areas should be tested. The mean value for each mask should be compared to the requirements. Both the tests for BFE and DP should be conducted on masks conditioned at 21 ± 5 °C and 85 ± 5% relative humidity for a minimum of 4 h before testing.

### 2.2. The Set-Up of Laboratory Facilities at the Universities in the Emergency Period

The following laboratories, among others in Italy, were activated during the emergency period and provided data for this study: “LASS-TN-Covid-19 Laboratorio Associato per la verifica di Dispositivi di Protezione, Dipartimento di Ingegneria Industriale Università di Trento and Laboratorio di Sanità Pubblica, Azienda Provinciale per i Servizi Sanitari di Trento” (UNITN), “Dipartimento di Ingegneria Enzo Ferrari, Università di Modena e Reggio Emilia and TPM” Science & Technology Park for Medicine, Mirandola (UNIMORE), “AntiCovidLab, Torre Biologica, Università degli Studi di Catania and INFN-Laboratori Nazionali del Sud, Catania” (UNICT), “Università degli Studi di Cagliari” (UNICA), “Lab. Microbiologia, Università di Camerino” (UNICAM), “Presidio Tecnico/Scientifico di Ateneo per l’Emergenza COVID-19, CESMA, Università di Napoli Federico II” (UNINA) and “LABC19 Centro di Ricerca e Servizio per l’Emergenza COVID-19, Università Politecnica delle Marche” (UNIVPM). All seven laboratories set up equipment for measuring DP and BFE, adhering as much as possible to the specifications of the standard EN 14683:2019+AC. Each installation had specific characteristics, which are described below, highlighting deviations from the standard.

**Figure 1:**
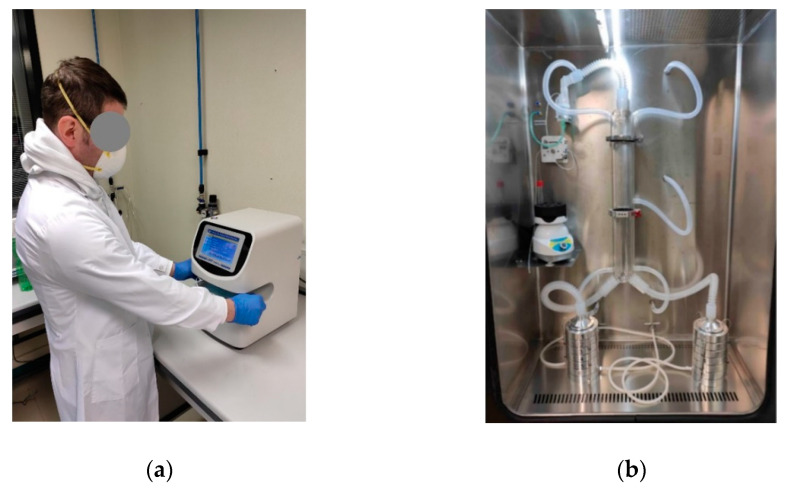
The test system for Differential Pressure (DP) (**a**) and Bacterial Filtration Efficiency (BFE) (**b**) at Università Politecnica delle Marche (UNIVPM) and Università di Camerino (UNICAM).

UNIVPM and UNICAM purchased and used a commercially available device (GBN701 face Mask Differential Pressure Tester, GBPI, China) (Figure 1a). It is a table-top compact system, fully integrated with a control unit, which performs the test sequence, controls the flow, measures the differential pressure and computes the specific pressure drop in Pa/cm^2^. Masks were clamped by a pneumatic actuator, which guaranteed the adequate sealing in order to avoid leaks. The system was calibrated on 14 April 2020 by the producer. During the series of measurements, it was not recalibrated. These laboratories also purchased a commercially available test system for BFE. Different from standard specifications, the system uses two six-stage Andersen impactors, connected in parallel downstream of the aerosol chamber through a Y junction (Figure 1b). This configuration allowed us to perform two tests simultaneously, or a test and a control. The system exploited two distinct vacuum pumps, each one generating the constant flow rate required by the standard. The air flow rate control was performed through a proportional–integral–derivative (PID) controller integrated in the vacuum pump. An optimization of the PID controller parameters was performed in order to keep flow rate oscillations below 1% of the set point. The aerosol was generated by a nebulizer and a peristaltic pump. The control of CFU count in positive controls was obtained by adjusting the test duration. CFUs were manually enumerated after 18 h of incubations to better discriminate closer colonies.

UNIMORE collaborated with Safe s.r.l. (Mirandola, Italy), a private laboratory specializing in biological and chemical analysis.

**Figure 2:**
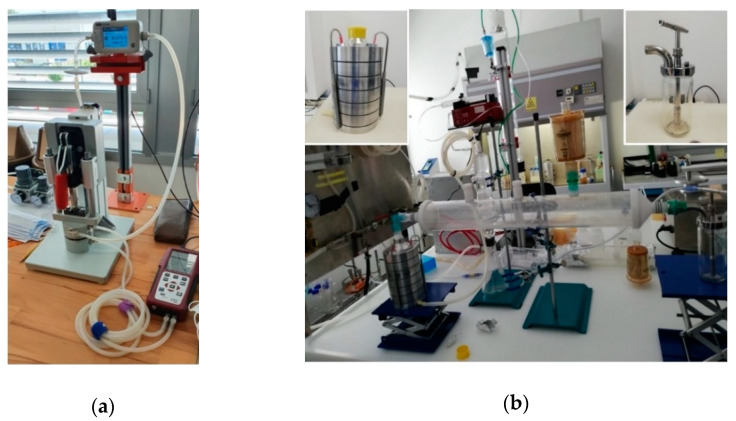
The test system for DP (**a**) and BFE (**b**) at Università di Modena e Reggio Emilia (UNIMORE). In the insets of the (**b**) image, the Andersen cascade impactor (top left) and the aerosol generator (top right) are shown.

For the DP measurement, the face mask sample was clamped through of a custom-made vise that allowed an adequate sealing (Figure 2a). A TSI 5200-2 Flowmeter (TSI Incorporated, Shoreview, MN, USA), calibrated on 5 December 2019, was used to set and monitor the air flow. A pressure gauge (MF plus, MRU Italia s.r.l., Thiene, Italy), calibrated on 18 February 2020, was used to collect the differential pressure measurement and to compute the specific pressure drop in Pa/cm^2^.

Tests for the BFE measurements were performed using a custom-made set-up (Figure 2b). The bacterial aerosol was generated by means of a collision nebulizer. The aerosol was then forced to enter into a 20 L glass cylinder serving as an expansion chamber, allowing the concentration of droplets to homogenize. At a constant flow rate, monitored and controlled through a TSI 5200-2 Flowmeter (TSI Incorporated, Shoreview, MN, USA) calibrated on 5 December 2019, the aerosol reached a six-stage Andersen impactor which was holding the face mask sample. Finally, an automatic colony counter was used to count the CFUs on agar plates.

**Figure 3:**
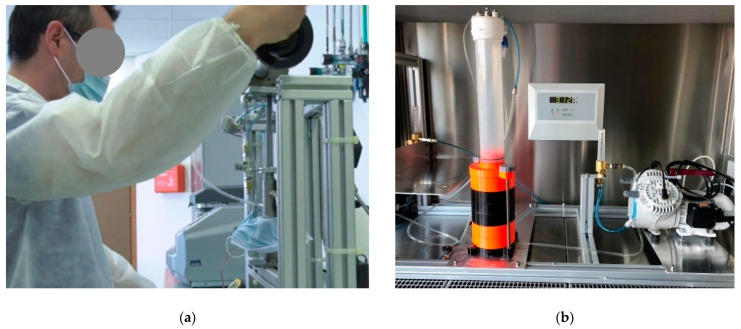
The test system for DP (**a**) and BFE (**b**) at Università degli Studi di Catania (UNICT).

The BFE and DP measurement systems at UNICT were created jointly with INFN-LNS, exploiting instrumentation and materials already available at the lab premises in order to minimize the commissioning time (Figure 3). The quantitative measurement of the DP was performed by means of a pressure sensor SDP816-500Pa (RS components, Milan, Italy). The 6-stage Andersen cascade impactor was manufactured by 3D printing of polymeric material in order to cope with time constraints and procurement difficulties caused by the lockdown. The design was based on the diagrams provided by Andersen [14].

The bacterial challenge was produced using the *Staphylococcus aureus* ATCC 25923 strain, equivalent in terms of genome size and antibiotic resistance profile to the *Staphylococcus aureus* ATCC 6538 indicated in the standard [16,17]. The manual colony count was carried out by performing a positive hole correction. A gold standard was not available for the assessment of operating conditions. However, two reference samples (named “Control A” and “Control B”) were used as internal controls. “Control A” and “Control B” showed BFE values in the 70–80% and 98.0–99.5% ranges, respectively, throughout the testing period. Each BFE measurement session was performed according to the following sequence: negative control, positive control, “Control A”, 2 test samples, “Control B”, 3 test samples, positive control. The analysis of 30 replicates of “Control B”, indicated an expanded uncertainty (99% confidence) of ±0.4% for BFE values above 95%.

**Figure 4:**
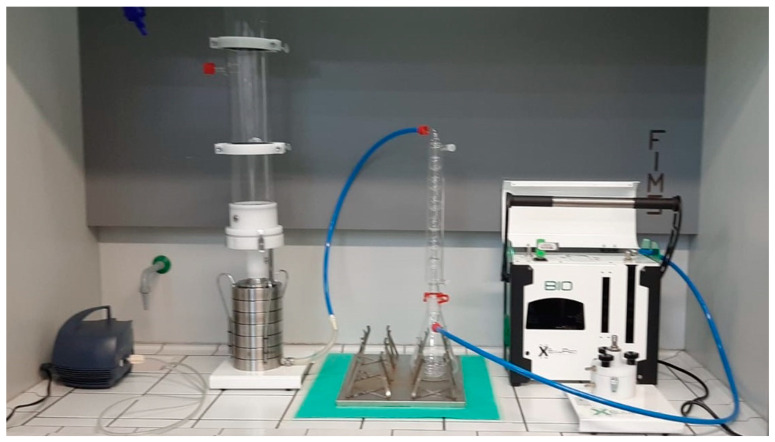
The test system for BFE and DP at Università degli Studi di Cagliari (UNICA).

UNICA purchased a commercially available test system from XEARPRO, Cogliate, Italy (Figure 4). The system allowed us to run all the measurements required by the standard for the verification of BFE and DP. It is composed by an electronic sampler (Bulldog X-Plus, XEARPRO, Cogliate, Italy), an aerosol chamber, a condenser, and a six-stage Andersen impactor. The bacterial aerosol was generated using a LC Sprint nebulizer (PARI GmbH, Starnberg, Germany).

**Figure 5:**
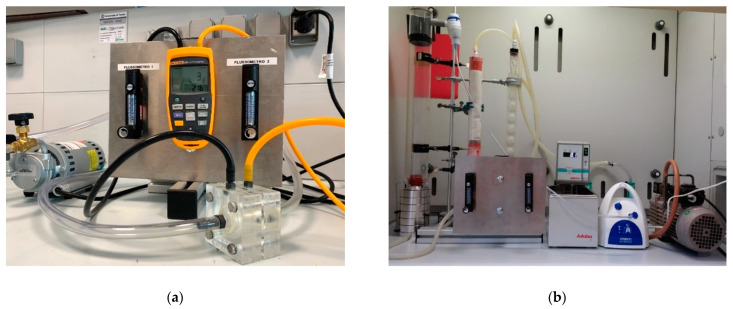
The test system for DP (**a**) and BFE (**b**) at Università di Trento (UNITN).

UNITN activated the LASS-TN-Covid-19, an associated laboratory for the assessment of personal protective devices, joining staff and structures available at the Department of Industrial Engineering and at the Public Health Laboratory of the Provincial Heath Trust of Trento Province. The set-up for the measurement of DP was custom-made (Figure 5a), according to specifications reported in the Annex C of EN 14683:2019+AC. The flow cell (a cylinder 25 mm in diameter and 32 mm high, made of two halves, each 16 mm high) was obtained by superimposing several polymethylmethacrylate slabs, laser cut and glued with epoxy resin. The 28 mm disk sample obtained from the mask was positioned between the two halves of the transparent flow chamber that were then pressed together using four screws and bolts. A 25 mm silicone elastomeric annular gasket prevented air leakage and forced the air flow through a sample circular section of 25 mm. The flow rate at 8 L/min was generated by a rotary pump and adjusted by two analog flow meters with control valves, positioned immediately before and after the flow cell. The pressure drop was measured by a digital airflow meter (Fluke 992, Fluke Corp., Everett, WA, USA), having a resolution of 1 Pa and calibrated by the manufacturer at the beginning of April 2020.

The set up for BFE measurement was made exploiting a six-stage Andersen impactor (Tisch six stage viable samples, Tisch Environmental Inc., Village of Cleves, OH, USA) and assembling the remaining part of the system according to the test rig indications given by the Annex B of EN 14683:2019+AC, with minor adaptations (Figure 5b). The bacterial aerosol was generated by a nebulizer (A3 Omron, Osaka, Japan), having a nominal mean diameter of 3 µm. The whole system was installed in a BSL-2 cabinet at the Public Health laboratory of the Provincial Health Trust of the Trento Province. Given the nebulizer consumption of microbial suspension (about 3 mL/min), higher than that recommended by the standard, the concentration of the bacterial suspension was reduced accordingly (@10^5^ CFU/mL) in order to be in line with the total CFUs on positive controls specified by the standard. No positive hole correction was applied to CFU counts, but CFUs on agar plates were enumerated manually after 15 h of incubation at 36 °C in order to discriminate between closer colonies. Each mask was tested in quintuplicate. A positive control (without the mask before the impactor) was obtained before and after testing the five samples. A negative control (without mask and without generating the microbial aerosol) was obtained at the end of each experimental session.

**Figure 6:**
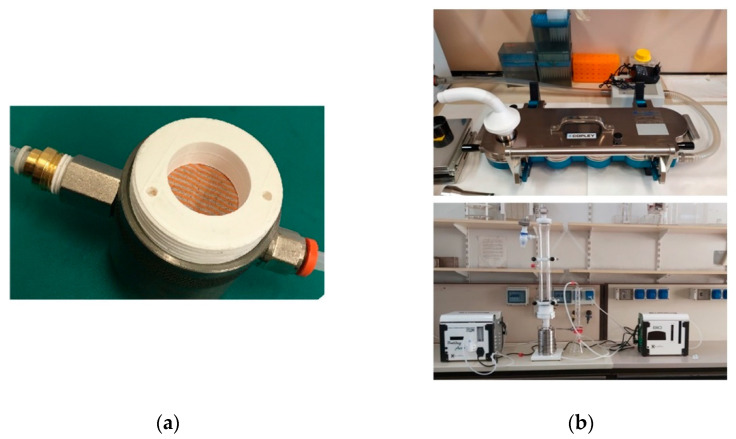
The test system for DP (**a**) and BFE with next generation impactor (**b**, top); BFE with Andersen cascade impactor (**b**, bottom) at Università di Napoli Federico II (UNINA).

At UNINA, the test rig for DP (Figure 6a) was custom-built using a cylindrical steel reactor in which the sealing cap was replaced with a sample holder. The sample holder, screwed to the reactor, consisted of two 3D-printed polymeric rings. One ring was in contact with the test cell through a sealing O-ring, the other was threaded and screwed to the test cell, binding the specimen. The air inlet and the pressure transducer were connected to the test cell. A differential pressure transducer (MKS 226, MKS Instruments Inc., Andover, MA, USA) with full scale of 10 torr and accuracy of 0.1% of the full scale was used for the measurement of DP, using an MKS PR400 reader. The air flow was controlled using a calibrated flow meter (ASA 2500, Milan, Italy). After sample conditioning, a blank measurement was obtained without the sample, then the disk/mask was positioned, and the DP was measured.

At an early stage, for BFE measurements, UNINA adopted a multistage impactor (Next Generation Impactor, NGI, Copley Scientific Ltd., Nottingham, UK) already available at their lab. The system is equivalent to Apparatus E of the European Pharmacopoeia for the aerodynamic assessment of fine particles of preparations for inhalation and it is shown in Figure 6b (top). The multistage impactor was characterized by seven stages (cups) and a micro-orifice collector arranged on a single plane. A flowmeter was placed between the impactor and the vacuum pump (DFM4, Copley Scientific, UK) to control air flow rate through the system. The bacterial aerosol was delivered through a nebulizer placed in series with the impactor. Each mask sample was placed in a 3D printed holder.

The bacterial challenge was prepared according to the standard specifications. The size and zeta potential of bacterial suspension were assessed by a particle sizer (nano ZS, Malvern Panalytical Ltd., Malvern, UK). Bacteria were recovered by washing the cups with peptone water, collected by centrifugation, placed in tryptic-soy agar and counted. The protocol consisted in running 5 positive controls, 2 negative controls and 5 assays on 5 mask specimens.

UNINA’s BFE tests were supported by two other filtration tests: a particle filtration test using oil particles, following the method reported in the standard EN 149:2009+A1 [18], and a fast screening of Aerosol Filtration Efficiency (AFE) without the use of bacteria. In the latter case, a rhodamine B (Rho) solution in peptone water was employed in the same experimental conditions described above [19]. This triad of tests provided a superior determination of mask filtration properties, covering a larger range of aerosol size (90 nm–14.1 µm) and reduced the uncertainties of the EN 14683:2019+AC methods related to the adoption of the BFE test alone. Modelling of the experiments was also performed to support the data analysis and provide a robust assessment of mask performances.

Later, the UNINA’s lab acquired the standard 6-stage Andersen Cascade Impactor, (TCR Tecora IMP-6BIO, Cogliate, Italy) developed in accordance with EN 14683 (Figure 6b, bottom). The BFE tests carried out with the NGI were replicated with the 6-stage Viable Cascade Impactor and results were consistent between the two methods [19].

## 3. Results and Discussion

### 3.1. Laboratories’ Activity Report

Immediately after the DPCM publication on 17 March 2020 [5], the Italian Universities participating in this study reacted to the emergency. The first lab was activated within a week.

**Figure 7:**
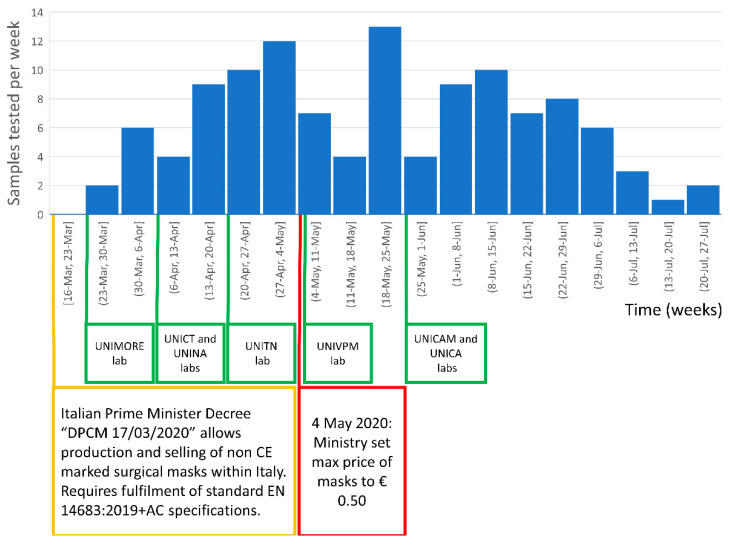
Laboratory response time and workload. Activation of the various university laboratories is indicated in the green boxes. The date of the Decree of the President of the Council of Ministers (DPCM) allowing the import, production and distribution of non-CE marked (conformity with health, safety, and environmental protection standards for products sold within the European Economic Area) surgical masks is indicated in the yellow box. In the red box, the date when the selling price was fixed to EUR 0.50 per mask is given.

In no more than 10 weeks, all the other labs were also operative and a collaborative network between laboratories was established. Figure 7 provides the chronological overview of the laboratory network settling and activity. The activation date of the different labs is indicated in green. The DPCM publication date is reported as a reference starting date, generating the demand for mask testing. In red is also reported the date of 4 May 2020, when the Italian Government set the mask price to max EUR 0.50 to limit market price fluctuations and possible speculations in order to guarantee the population’s access to masks [20]. The number of samples tested per week by the whole lab network is also shown. Only data referring to fully tested sample (five BFE and five DP measurements performed per each mask/prototype) were included in this study and are displayed in the bar chart of Figure 7.

A total of 120 masks/prototypes underwent the full set of BFE and DP tests according to EN 14683:2019+AC in the period from 23 March to 24 July 2020. The workload of the laboratory network reached a maximum of 13 masks/prototypes per week in the period from 18 May to 25 May 2020. Laboratories’ activities were suspended or reduced to a minimum after the last week of July due to the summer holiday period. In September, activities started again at a markedly slower rate (data not shown).

The limitation to the selling price had a significant impact on the market, changing the type of masks and the characteristics of the manufacturers/importers. Possibly, this was also the reason for the apparent decrease in the number of testing requests at the beginning of May 2020. The number of requests for testing was again elevated in June 2020.

### 3.2. Mask Performances

**Figure 8:**
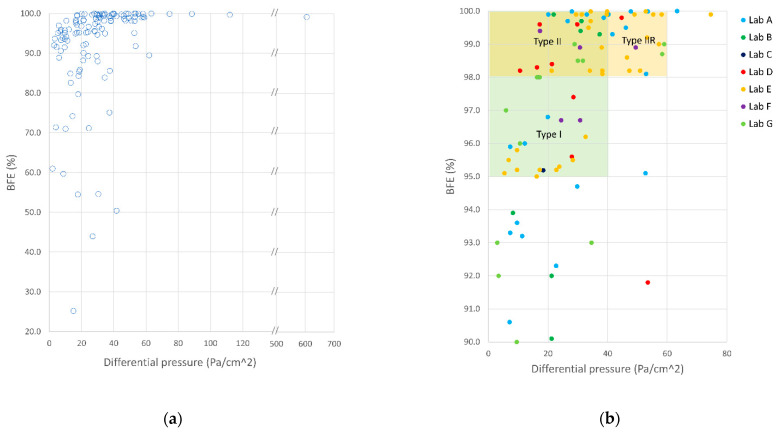
Bacterial filtration efficiency (BFE) vs. breathability (DP). All collected data are presented in the left graph (**a**). The right graph (**b**) presents a subset of data closer to the highest performance. Ranges of performance required by EN 14683:2019+AC are indicated for Type I, Type II and Type IIR masks. Data are color-coded by laboratory.

Ideal mask performance should tend to 100% BFE and 0 Pa/cm^2^ DP, thus allowing both optimal filtration and breathability. All collected data (referring to 120 tested masks) are presented in the BFE vs. DP scatter graph of Figure 8a. DP and BFE data for the tested items ranged from 2.0 to 605.1 Pa/cm^2^ and from 25.2 to 100.0%, respectively.

The ideal combination of BFE and DP performance, represented by the upper-left corner of the graph, was not achieved by any of the tested samples.

The right scatter plot in Figure 8b presents a subset of data closer to the highest performance. For ease of comparison, areas with acceptable performances for Type I, Type II, and Type IIR surgical masks are also indicated (see Table 1).

A quantitative analysis of these results reveals that 54 (45.0%), 34 (28.3%) and 53 (44.2%) out of the 120 tested masks satisfied the performance requirements for BFE and DP set in the standard for Type I, Type II and Type IIR surgical masks, respectively. Overall, 73 (60.8%) out of the 120 tested masks possessed BFE and DP parameters suitable for surgical masks. The remaining 47 (39.2%) masks were considered as possible community masks. It is interesting to observe that there is no apparent clustering in sample performance for tests carried out in one or more specific laboratories. This suggests that each lab was asked to test a variety of samples that resulted in a wide range of performance values.

**Figure 9:**
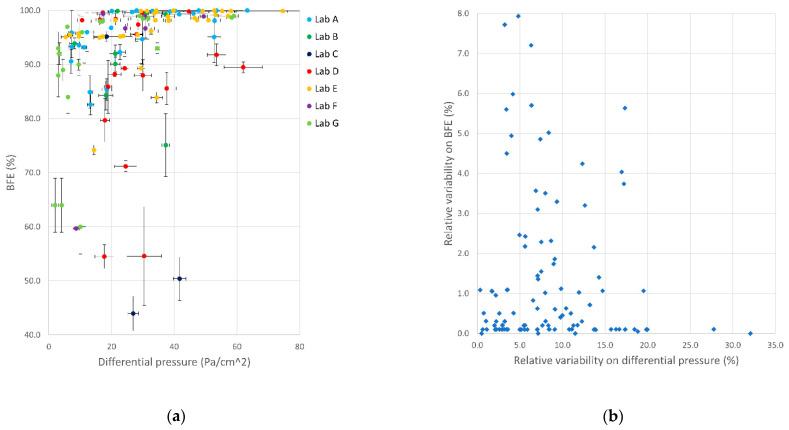
Variability of BFE and DP measurements among the five samples of each mask model/prototype tested by the whole laboratory network (**a**) BFE vs. DP data showing standard deviations as error bars in a subset of data; (**b**) relative variability (error percentages over five measurements) on BFE vs. relative variability of DP measurements in the whole mask dataset.

The variability of BFE and DP measurements is depicted in Figure 9. The error bars (vertical and horizontal) in the scatter graph of Figure 9a represent standard deviations calculated on both DP and BFE over five samples of the same mask model/prototype. These intervals include both measuring uncertainty and sample variability. The measurement uncertainty was generally lower than the variability due to differences among the five tested samples.

Figure 9b shows the relative percentage variability on DP and BFE data over the full dataset, and accentuates that no significant correlation was present between these two terms. The relative percentage variability on DP measurements was higher (median 7.3%, interquartile range 8.6%) than that on BFE measurements (median 0.4%, interquartile range 1.5%). High values of BFE—namely, close to 100%—were typically associated with lower variability of the measurements. On the contrary, low BFE measurements (typically below 80%) suffered from high variability among samples of the same mask.

Table 2 and Table 3 summarize the available information about mask material, mask design (in terms of number of layers), and fiber structure. The large majority of the tested masks, 77 (64.1%) out of 120, were made entirely of polypropylene. Masks produced using only polyamide, polyester, or cellulose accounted, respectively, for only 2 (1.7%), 1 (0.4%), and 1 (0.4%) samples. Masks composed of two or more materials totaled 24 (20%). Among these, 12 masks included natural materials such as cotton or cellulose.

The most frequently encountered fiber structure was nonwoven (89 masks), including both spunbonded and meltblown sheets. On the contrary, masks composed of only woven or knitted fabrics were only seven (5.8%). Fabrics were also used in 11 (9.2%) additional masks composed of both woven and nonwoven fibers, where the major filtration effect was typically obtained, including a meltblown polypropylene sheet in between the fabric layers.

**Figure 10:**
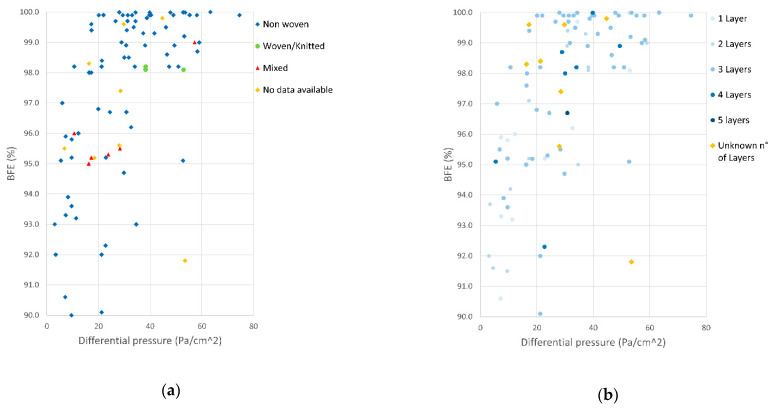
Mask performance grouped by fiber structure of the layers (**a**) and by number of layers (**b**).

Figure 10 displays the performance of the tested masks by their materials (Figure 10a) or by their number of layers (Figure 10b). Both graphs present the subset of BFE and DP data close to the highest mask performance. In the first period following the promulgation of the DPCM, masks arriving to laboratories were typically made of woven/knitted materials produced by local textile companies that converted their productions to comply with the pandemic need and market demand. Once the import activities of goods from abroad (China in particular) were restored, masks made with nonwoven layers were once again the majority of the tested items.

The masks with the highest combined performance, among those tested, were invariably made of three layers. In addition, the majority of the masks with values of BFE >99% were manufactured using combinations of nonwoven spunbonded and meltblown polypropylene layers. These manufacturing characteristics are those typical of the spunbonded–meltblown–spunbonded triple layer polypropylene masks, also available before the COVID-19 pandemic, thus representing the typical surgical mask composition and design.

The presence of a meltblown polypropylene layer within the layers of the mask was usually associated with good performances at the BFE test. Masks composed using fewer than three layers (light blue dots in Figure 10b) were often made by only single or multiple layers of spunbonded polypropylene and showed better breathability, but bacterial filtration efficiency below the thresholds indicated by the standard. All masks/prototypes made by only woven/knitted materials included pure cotton, cotton/artificial fibers (polyester, polyurethane), and only artificial fibers (polyamide, polyester). These kinds of masks showed poor performances usually in terms of both BFE and DP, or at least in one of the two parameters (green dots in Figure 10a). These findings are in agreement with previously published data on community masks made of fabrics [21,22,23] and should be considered in a comprehensive evaluation of mask performance. In fact, despite no clear effect on blood or muscle oxygenation being found in healthy subjects wearing a face mask [24], low breathability can have an impact on the user’s comfort [23] and may contribute to incorrect use in the general population [25,26]. Therefore, very low values of DP (e.g., <20 Pa/cm^2^) could be considered an added value for increasing the willingness of the population to wear masks where no high level of contaminated aerosol is expected (e.g., outdoor areas or other areas where community masks are recommended). In summary, none of the new proposed materials and designs were able to outperform the typical polypropylene surgical masks present in the market before the pandemic.

### 3.3. Laboratories’ Adherence to Standards Specifications

**Figure 11:**
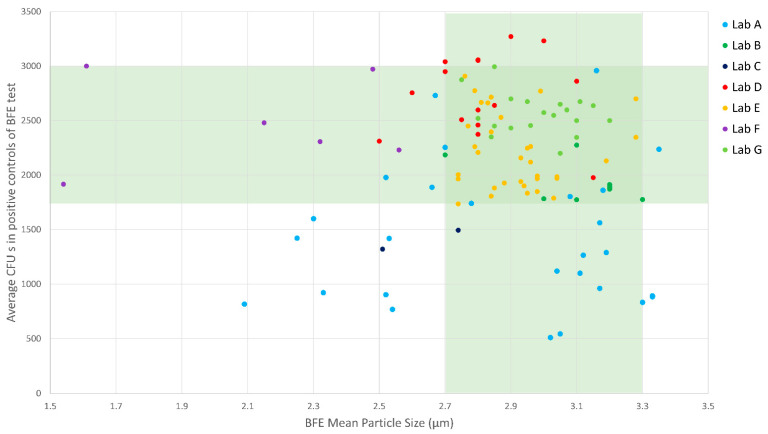
Lab adherence to measurement requirements. Light green bands identify the correct measuring requirement for mean particle size (MPS) or total Colony Forming Units (CFUs) of the positive controls. The dark green area corresponds to measurement performed adhering to both MPS and CFUs requirements. Data are color-coded by laboratory.

The adherence of laboratories was monitored and analyzed collecting the two main measurement specifications indicated in the standard: the mean particle size (MPS) of the aerosol and the average number of CFUs in the positive control of the BFE test (*CFU_control_*). Data from the whole laboratory network are reported in Figure 11. The vertical and horizontal light green bands identify the correct range for the MPS and for the CFUs of the positive control, respectively. The dark green area corresponds to measurements carried out adhering to both MPS and CFUs requirements. A total of 74 (61.7%) out of the 120 tested masks were analyzed, with both MPS and total CFUs in positive controls being in accordance with EN 14683:2019+AC specifications. A total of 110 testing procedures (91.7%) (out of the 120 masks) satisfied at least one of the two specifications. Considering that all seven laboratories set up completely new laboratory facilities and the relative measurement procedures in a very limited timeframe, operating in an emergency situation, the rate of adherence to the requirements of the standard was considered adequate. Mean particle size (MPS) appears to be related to the lab (data clustered according to lab color coding in Figure 11). This could be related to several factors such as differences of aerosol nebulizer among laboratories, differences in the design and aerosol flow path in between the aerosol generator and the multistage cascade impactor, difficulty in tuning the custom-made set-ups, etc. The MPS distribution is also skewed towards lower values, which possibly resulted in a more challenging test for BFE given that smaller droplets are expected to be filtered less effectively.

A similar skewed distribution was observed also for the values of total CFUs in positive controls. A total of 19 (15.8%) tests out of 120 resulted in a total CFU lower than 1700. However, no test was performed with fewer than 500 CFUs on positive controls. Values lower than 500 CFUs significantly affect the resolution and reliability of the BFE measurement. Indeed, a test performed with a colony count lower than 500 CFUs in positive controls cannot provide BFE values with a resolution better than 0.2%, which corresponds to an expanded uncertainty of about 0.4% on the BFE value. This uncertainty can limit the correct association of mask to standard type or even the check for mask conformity to standard requirements for BFE.

### 3.4. The Organizational Strategies: An Example of University Third Mission in an Emergency

The sequence of tests specified by the EN 14683:2019+AC standard is complex and rather time consuming. However, the breathability test is by far the quickest to be performed. Therefore, all laboratories have implemented some procedure to provide a prompt response to the demand for testing coming from the manufacturers/importers. Three different strategies were adopted by the different labs participating in this study:

1. two step procedure (UNICAM, UNIVPM, UNIMORE, UNITN, UNICA), performing tests in sequence, starting from DP test, and proceeding to BFE with a 6-stage Andersen impactor only if DP requirements were satisfied;
2. three step procedure with simplified BFE (UNICT), performing tests in sequence, starting from DP test, and proceeding to a simplified BFE (impactor with reduced number of stages or full number of stages, but no sample replicates) only if DP requirements were satisfied, then proceeding to a complete BFE analysis with the 6-stage Andersen impactor only in case the simplified BFE provides a positive result;
3. four step procedure with cross-check testing (UNINA), performing tests in go/no go stages. Stage 1: measure of DP and particle filtration; Stage 2: Aerosol filtration efficiency; Stage 3: Complete BFE, Stage 4: other testing for conformity to EN 14683:2019+AC (e.g., microbial load, splash resistance) and EN 10993-1:2010 (biocompatibility tests).

This approach was a clear example of implementation of the so-called university “Third Mission” [27,28] in an emergency context, which required concentrating the limited resources (time, personnel and equipment) on the more promising prototypes, also keeping the other two mandates represented by teaching/education and research activities active.

To manage the large demand for testing, each university implemented an internal organization, whose main pillars have been a dedicated laboratory and a website [29,30,31,32,33,34,35], sometimes with the support of the technology transfer unit. The researchers have been called not only to perform tests, but also to provide consultancy, information and assistance to the private companies who were facing the challenge to reach the compromise between breathability and filtration efficiency required by the standard. Such an action represents one form of technology transfer as well as a form of societal engagement, both elements of the “Third Mission” of the university as described in the literature [27,28].

It is worth noticing that each laboratory has received a large number of applications from companies: altogether, the seven laboratories had ~600 contacts between May and July 2020, 65% of these were from small-medium enterprises, in prevalence from the textile sector. However, less than 40% of them resulted in contracts for testing and only 120 were fully tested according to EN 14683:2019+AC for DP and BFE, while the others stopped after failing the preliminary DP or BFE test or because the producer was not interested in testing the full performance parameters of the masks (e.g., for masks dedicated to community use). These figures most properly depict the overall impact of this emergency action.

## 4. Conclusions

The paper reports the timely reaction of a network of seven Italian universities to the need of setting up a measurement infrastructure in support of the production and import of surgical masks to face the first wave of the COVID-19 pandemic in Italy, in the period from March to July 2020. This represented a tangible example of the “Third Mission” of the university, particularly relevant for its impact during an emergency.

Each university reacted and set up laboratories by following different strategies, either by designing/adapting/building new equipment or by purchasing and tuning specific test equipment. All laboratories operated as much as possible in agreement with the methods reported for DP and BFE in the EN 14683:2019+AC standard and were able to satisfy the large majority of the testing request and method specifications, despite working in an emergency context. The laboratories became operative in less than one month from the demand onset.

During the emergency, turnaround time, human resources and acquisition of equipment and consumables were a concern. In order to deal with the high demand for testing and maximizing the productivity, each laboratory identified its own strategy for optimizing the tests procedures and maximizing response efficiency. Most frequently, prioritization in testing was associated with products showing better chances to fulfill the requirements. In most cases, a first screening was based on DP measurement and/or simplified BFE tests.

Overall, in less than three months, the network of laboratories characterized 120 mask models, following the complete test sequence for BFE and DP according to standard EN 14683:2019+AC. Most of the masks were produced in Italy by companies that entered this market for the first time, while a limited number was imported. Support and consultancy to the manufacturers in the self-certification process and in the production of the relevant documentation was also provided.

The dataset displayed in this paper provides a snapshot of the Italian productive system performance in reacting to the pandemic. Notwithstanding the extremely challenging period, a significant percentage (60.8%) of the tested masks exhibited performances within the requirements for DP and BFE stated by the EN 14683+AC standard for Type I, Type II or Type IIR masks. The remaining ones were usually suitable to be used as community masks.

DP and BFE results indicated that masks made of nonwoven polypropylene, with at least three layers (spunbonded–meltblown–spunbonded) were still the optimal solution to ensure both good breathability and sufficient bacterial filtration. According to the data collected, this is actually the state of art design for surgery masks. Other tested fabrics and/or new materials/designs did not outperform polypropylene masks and were frequently associated with insufficient performance for one or both parameters, thus requiring more research and development to achieve better performance solutions.

The analysis of the data suggested an acceptable interlaboratory reproducibility of the BFE and DP measurements. Although some differences in adhering to methodological specifications among laboratories were present, this did not result in evident clustering or biasing of the BFE and DP values. This indicated that test procedures at each single lab achieved sufficient reliability, despite the short time for setting up and tuning the equipment and training the personnel. As a follow up of this work, a Round-Robin interlaboratory study has been planned and is currently running to better address intra- and interlaboratory measurement reproducibility.

## Figures and Tables

**Table 1:** Performance requirements for medical face masks for bacterial filtration efficiency (BFE) and differential pressure (DP) according to the standard EN 14683:2019+AC “*Medical face masks—Requirements and test methods*” [11].

Mask Type	Bacterial Filtration Efficiency (BFE) %	Differential Pressure (DP) Pa/cm^2^
I	≥95	<40
II	≥98	<40
II R	≥98	<60

**Table 2:** Number of tested masks broken down according to mask material and number of layers.

Mask Material ^1^	Mask Design					
	1 Layer	2 Layers	3 Layers	4 Layers	5 Layers	No. of Layers N.A. ^2^
Polypropylene	11	15	42	7	2	2
Polyamide	2	-	-	-	-	-
Polyester	-	-	1	-	-	-
Cellulose	-	1	-	-	-	-
Polypropylene/Polyester	-	-	6	-	-	-
Polypropylene/Polyurethane	-	-	1	-	-	-
Polypropylene/Polyamide	-	-	1	-	-	-
Polypropylene/Polyethylene	-	-	1	-	-	-
Polypropylene/Cotton	1	-	3	-	-	-
Polypropylene/Polyamide/Cotton	-	-	1	-	-	-
Polypropylene/Polyester/Cotton	-	-	1	-	-	-
Polyamide/polyurethane	1	-	-	-	-	-
Polyamide/Polypropylene/Cotton	-	-	2	-	-	-
Polyester/Polyurethane	-	1	-	-	-	-
Polyester/Cotton	-	1	1	-	-	-
Polyester/Cellulose	1	-	-	-	-	-
Polyurethane/Cotton	-	2	-	-	-	-
Material N.A. ^2^	-	1	1	-	-	11

^1^ Material of the sole filtering part. Ear laces, scaffolds, etc. are not considered here. ^2^ Information not available.

**Table 3:** Number of tested masks broken down according to the fiber structure.

Fiber Structure ^1^	Number of Masks
Nonwoven	89
Woven/Knitted	7
Mix of woven/fabric and nonwoven layers	11
Structure information N.A. ^2^	13

^1^ Structure of the sole filtering part. Ear laces, scaffolds, etc. are not considered here. ^2^ Information not available.

## Data Availability

The data presented in this study are available on reasonable request from the corresponding author. Part of the data are not publicly available due to contractual obligations with masks manufacturers.

## References

[1] Advice on the Use of Masks in the Community, during Home Care and in Healthcare Settings in the Context of the Novel Coronavirus (COVID-19) Outbreak. https://www.who.int/publications-detail-redirect/advice-on-the-use-of-masks-in-the-community-during-home-care-and-in-healthcare-settings-in-the-context-of-the-novel-coronavirus-(2019-ncov)-outbreak.

[2] (2020). CEN Workshop Agreement CWA 17553:2020 E, “Community Face Coverings—Guide to Minimum Requirements, Methods of Testing and Use”.

[3] European Center for Disease Prevention and Control Using Face Masks in the Community, ECDC-European Center for Disease Prevention and Control, Technical Report. https://www.ecdc.europa.eu/en/publications-data/using-face-masks-community-reducing-covid-19-transmission.

[4] Gazzetta Ufficiale dell Repubblica Italiana (2020). Decreto Legge n.9 Del 2 Marzo 2020 (Art. 34, Comma 3).

[5] Gazzetta Ufficiale dell Repubblica Italiana (2020). Decreto Legge Del 17 Marzo 2020 n.18 (Art. 15).

[6] World Health Organization Rational Use of Personal Protective Equipment for Coronavirus Disease (COVID-19) and Considerations during Severe Shortages—Interim Guidance 2020. https://www.who.int/publications/i/item/rational-use-of-personal-protective-equipment-for-coronavirus-disease-(covid-19)-and-considerations-during-severe-shortages.

[7] The Organisation for Economic Co-operation and Development The Face Mask Global Value Chain in the COVID-19 Outbreak: Evidence and Policy Lessons. http://www.oecd.org/coronavirus/policy-responses/the-face-mask-global-value-chain-in-the-covid-19-outbreak-evidence-and-policy-lessons-a4df866d/.

[8] Yu J., Lu Y., Muñoz-Justicia J. (2020). Analyzing Spanish News Frames on Twitter during COVID-19—A Network Study of El País and El Mundo. Int. J. Environ. Res. Public Health.

[9] Ahmed W., Vidal-Alaball J., Lopez Segui F., Moreno-Sánchez P.A. (2020). A Social Network Analysis of Tweets Related to Masks during the COVID-19 Pandemic. Int. J. Environ. Res. Public Health.

[10] Istituto Superiore di Sanità Procedure per Richiesta Produzione Mascherine—ISS. https://www.iss.it/procedure-per-richiesta-produzione-mascherine.

[11] European Committee for Standardization (2019). EN 14683:2019+AC Medical Face Masks—Requirements and Test Methods 2019.

[12] International Standard Organization (2018). ISO 10993-1:2018 Biological Evaluation of Medical Devices—Part 1: Evaluation and Testing within a Risk Management Process 2018.

[13] Marple V.A. (2004). History of Impactors—The First 110 Years. Aerosol Sci. Technol..

[14] Andersen A.A. (1958). New Sampler for the Collection, Sizing, and Enumeration of Viable Airborn Particles. J. Bacteriol..

[15] Macii D., Petri D. (2009). Guidelines to Manage Measurement Uncertainty in Conformance Testing Procedures. IEEE Trans. Instrum. Meas..

[16] del Wacher-Rodarte M.C., Trejo-Muñúzuri T.P., Montiel-Aguirre J.F., Drago-Serrano M.E., Gutiérrez-Lucas R.L., Castañeda-Sánchez J.I., Sainz-Espuñes T. (2016). Antibiotic Resistance and Multidrug-Resistant Efflux Pumps Expression in Lactic Acid Bacteria Isolated from Pozol, a Nonalcoholic Mayan Maize Fermented Beverage. Food Sci. Nutr..

[17] Treangen T.J., Maybank R.A., Enke S., Friss M.B., Diviak L.F., Karaolis D.K.R., Koren S., Ondov B., Phillippy A.M., Bergman N.H. (2014). Complete Genome Sequence of the Quality Control Strain Staphylococcus Aureus Subsp. Aureus ATCC 25923. Genome Announc..

[18] European Committee for Standardization (2009). EN 149:2009+A1 Respiratory Protective Devices—Filtering Half Masks to Protect against Particles—Requirements, Testing, Marking 2009.

[19] D’Anna A., Di Natale F., De Falco G., Di Maio E., Tammaro D., Quaglia F., Ungaro F., Cassiano C., Salvatore P., Colicchio R. (2020). Validazione di maschere chirurgiche nella fase di emergenza COVID19: L’esperienza dell’Università degli Studi di Napoli Federico II. G. Ital. Med. Lav. Ergon.

[20] Arcuri D.D. (2020). Ordinanza n. 11 /2020.

[21] Aydin O., Emon B., Cheng S., Hong L., Chamorro L.P., Saif M.T.A. (2020). Performance of Fabrics for Home-Made Masks against the Spread of COVID-19 through Droplets: A Quantitative Mechanistic Study. Extreme Mech. Lett..

[22] Shakya K.M., Noyes A., Kallin R., Peltier R.E. (2017). Evaluating the Efficacy of Cloth Facemasks in Reducing Particulate Matter Exposure. J. Expo. Sci. Environ. Epidemiol..

[23] Lee K.-P., Yip J., Kan C.-W., Chiou J.-C., Yung K.-F. (2020). Reusable Face Masks as Alternative for Disposable Medical Masks: Factors That Affect Their Wear-Comfort. Int. J. Environ. Res. Public Health.

[24] Shaw K., Butcher S., Ko J., Zello G.A., Chilibeck P.D. (2020). Wearing of Cloth or Disposable Surgical Face Masks Has No Effect on Vigorous Exercise Performance in Healthy Individuals. Int. J. Environ. Res. Public Health.

[25] Machida M., Nakamura I., Saito R., Nakaya T., Hanibuchi T., Takamiya T., Odagiri Y., Fukushima N., Kikuchi H., Amagasa S. (2020). Incorrect Use of Face Masks during the Current COVID-19 Pandemic among the General Public in Japan. Int. J. Environ. Res. Public Health.

[26] Khubchandani J., Saiki D., Kandiah J. (2020). Masks, Gloves, and the COVID-19 Pandemic: Rapid Assessment of Public Behaviors in the United States. Epidemiologia.

[27] Compagnucci L., Spigarelli F. (2020). The Third Mission of the University: A Systematic Literature Review on Potentials and Constraints. Technol. Forecast. Soc. Chang..

[28] Berghaeuser H., Hoelscher M. (2020). Reinventing the Third Mission of Higher Education in Germany: Political Frameworks and Universities’ Reactions. Tert. Educ. Manag..

[29] Lass-Tn-Covid-19, Progetto Mascherine, Dipartimento Di Ingegneria Industriale, Università Di Trento. https://www.dii.unitn.it/1208/progetto-mascherine.

[30] LABC19—Centro di ricerca e servizio per l’emergenza Covid-19—Università Politecnica delle Marche. https://labc19.univpm.org/.

[31] Tecnopolo Mario Veronesi Mirandola. https://tpm.bio/.

[32] Safe S.r.l, Analisi Chimico Biologich. https://www.laboratoriosafe.it/.

[33] Anti_Covid-Lab, Laboratorio per Il Test Di Tessuti per Dpi, Università Degli Studi Di Catania. http://www.brit.unict.it/.

[34] Laboratorio Emergenza Covid-19—Laboratorio DPI, Center of Advanced Metrology and Technology Services, Università di Napoli Federico II. http://www.cesma.unina.it/laboratori/presidio-tecnico-scientifico-per-l-emergenza-covid-19/presidio-tecnico-scientifico-per-l-emergenza-covid-19-laboratori-aree-di-intervento/laboratorio-emergenza-covid-19-laboratorio-dpi.

[35] Laboratorio Di Certificazione Delle Mascherine, Area Ricerca, Trasferimento Tecnologico e Gestione Progetti Università Di Camerino. https://aripro.unicam.it/content/laboratorio-di-certificazione-delle-mascherine.

